# Supplementary material for: The genome sequence of the Deathwatch beetle, Xestobium rufovillosum (De Geer, 1774)
Source: Wellcome Open Res. 2024 Oct 21;9:619. [Version 1] doi: 10.12688/wellcomeopenres.23210.1 (PMC12504945; doi:10.12688/wellcomeopenres.23210.1)
Supplement: Peer review history

# Reviewer report: Reviewer response for version 1

**DOI:** 10.21956/wellcomeopenres.25562.r133739

**Published:** 2025-10-27

**Authors:** Terrence Sylvester

**Affiliations:** 1 The University of Memphis, Memphis, Tennessee, USA

The authors present the genome sequence of the Deathwatch beetle, *Xestobium rufovillosum*. The background section is particularly engaging, providing both historical and biological context that captures the species’ ecological and cultural relevance. The authors effectively describe the origin of the beetle’s name, its wood-boring behavior, association with wood-decaying fungi, and the potential role of odorant receptors in host selection. This introduction not only situates the species within its ecological niche but also underscores the broader significance of this genome for understanding wood-feeding adaptations and pest biology.

 The methods are clearly and effectively described. The authors follow a standard and logical workflow. The sequencing depth appears sufficient and supports the generation of a high-quality assembly.

 The resulting genome is of excellent quality, as demonstrated by standard assessment metrics. Overall, this assembly represents a valuable genomic resource for exploring the evolution and molecular basis of wood-feeding in beetles.

Are sufficient details of methods and materials provided to allow replication by others?

Yes

Is the rationale for creating the dataset(s) clearly described?

Yes

Are the datasets clearly presented in a useable and accessible format?

Yes

Are the protocols appropriate and is the work technically sound?

Yes

Reviewer Expertise:

Genomics

I confirm that I have read this submission and believe that I have an appropriate level of expertise to confirm that it is of an acceptable scientific standard.

# Reviewer report: Reviewer response for version 1

**DOI:** 10.21956/wellcomeopenres.25562.r133737

**Published:** 2025-10-07

**Authors:** Chenyang Cai

**Affiliations:** 1 Chinese Academy of Sciences, Beijing, Beijing, China

This manuscript presents a high-quality genome assembly of the Deathwatch beetle *Xestobium rufovillosum*, an ecologically and economically important wood-boring beetle with cultural and historical relevance due to its association with historic buildings and ships, such as HMS Victory. The study is part of the Darwin Tree of Life project and follows their standardized workflow for specimen acquisition, sequencing, assembly, and curation.

 This manuscript is well-structured, and provides an important genomic resource for future research on beetle evolution, wood-feeding adaptations, pest control. The genome assembly is of chromosome-level completeness, with excellent metrics (BUSCO completeness 99.3% and the mitochondrial genome is also provided. The methods are described in great detail and include transparent data processing and curation pipelines. The manuscript is appropriate for publication as a genome report.  It is better to list the subfamily which the species belongs to.

Are sufficient details of methods and materials provided to allow replication by others?

Yes

Is the rationale for creating the dataset(s) clearly described?

Yes

Are the datasets clearly presented in a useable and accessible format?

Yes

Are the protocols appropriate and is the work technically sound?

Yes

Reviewer Expertise:

beetles. genomics

I confirm that I have read this submission and believe that I have an appropriate level of expertise to confirm that it is of an acceptable scientific standard.

# Reviewer report: Reviewer response for version 1

**DOI:** 10.21956/wellcomeopenres.25562.r108066

**Published:** 2024-11-07

**Authors:** Arun Arumugaperumal

**Affiliations:** 1 Department of Biotechnology, Rajalakshmi Engineering College, Chennai, Tamil Nadu, India

This is the first published article on the genome sequence of the Deathwatch beetle *Xestobium rufovillosum*. The genome assembly size was reported as 475.5 Mb. The mitogenome was also assembled and the length was 19.9 kb. The methodology used was very apt to the aim of the data article. The genome sequence was evaluated to be a near-complete one. This resource can be used to carry out further research on the organism. 

 The authors could have given a panel of different views of the specimen instead of a single photograph. The authors could have added a note on the number of protein-coding genes predicted. It was mentioned that there were some symbiotic bacteria and other organism sequences. A link to the data associated with those sequences could be useful for readers in taking forward the research about this beetle.

Are sufficient details of methods and materials provided to allow replication by others?

Yes

Is the rationale for creating the dataset(s) clearly described?

Yes

Are the datasets clearly presented in a useable and accessible format?

Yes

Are the protocols appropriate and is the work technically sound?

Yes

Reviewer Expertise:

Bioinformatics, Genomics

I confirm that I have read this submission and believe that I have an appropriate level of expertise to confirm that it is of an acceptable scientific standard.
